# General Medicine and Therapeutics

**Published:** 1895-03

**Authors:** 


### GENERAL MEDICINE AND THERAPEUTICS.

EDITED BY DR. LUTHER B. GRANDY.

### THE SERUM TREATMENT OF DIPHTHERIA IN AMERICA.

Up to the present, the number of cases of diphtheria reported from America as having been treated with antitoxin injections is not very large, but the observers who have used it seem to agree that it modifies very considerably the course of the disease, and rapidly produces a beneficial effect on the general condition. The mortality statistics, so far as they have been published, seem to indicate a distinct diminution in the death-rate, especially if the treatment is commenced early. There are very few accounts of any ill effects following the injections, and these were chiefly insignificant urticarial eruptions.

Dr. Arnold W. Catlin,[1] of Brooklyn, reports the case of a child, nine years of age, who was seen on the second day, and diagnosed as a case of diphtheria, the diagnosis being verified by a culture of the bacilli from the membrane by the New York board of health. Five grams of the antitoxin (Aronson's) were injected at one time between the shoulders. The child was no better for thirty-six hours, then began to improve, the temperature falling, the membrane disappearing, and general condition becoming much better. Bacilli were found in cultures from the throat for twenty-nine days, though there was no evidence of the disease six days after the use of antitoxin. The author considers the presence of bacilli so long after the disease was apparently cured emphasizes the wisdom of making cultures and quarantining patients as long as bacilli are found.

Dr. Geo. A. Muehleck,[2] of Philadelphia, reports six cases of diphtheria, in four of which antitoxin was used in doses not exceeding in any case 1½ cc. of Schering's antitoxin. In three out of the four cases the diagnosis was confirmed bacteriologically by Dr. Ravenel. After each injection Dr. Muehleck noticed a rapid fall in temperature, which rose again in twenty-four hours, but never so high as before injection. There was marked amelioration as regards the difficulty of breathing. The heart was less favorably influenced, being often irregular and weak. The membranes did not seem to thrive, failed to spread, and in twenty-four to forty-eight hours assumed a fatty pultaceous appearance, disappearing within a week. He does not think the cases were mild ones, as two died which could not be treated with antitoxin, owing to there being none available, whilst the other four recovered.

Dr. Augustus Caillé[3] gives nine cases treated with Aronson's antitoxin in doses of 2 cc. injected into the anterior part of the thigh. In all cases but one the diagnosis of true diphtheria was established by the board of health of New York City, and in this one case notification met with no response. His first case was seen only two hours before death, and he therefore excludes it from his statistics, which show, then, a mortality of one in eight. All the cases were of a severe type. Only one case was seen at onset, and this recovered rapidly after two injections. All the other cases were advanced, and supplementary treatment was employed. A fall of temperature was noted after injection, and re-establishment of sleep and appetite. No untoward effects occurred. Membrane disappeared, and periglandular infiltration subsided rapidly.

Dr. A. Campbell White,[4] at the meeting of the New York Academy of Medicine, drew attention to the results of the treatment at the Willard Parker Hospital. Twenty selected cases of a very malignant type were treated, the average age being 3.5 years, and the average dose of Aronson's strong serum 11.3 cc. Rashes on skin followed injections in three cases, diagnosed in one case as scarlet fever, in another measles, the third being a transient rash. Fourteen cases had laryngeal complications, four requiring intubation and one tracheotomy. One intubation case died on twenty-fourth day from lobular pneumonia, the tracheotomy case died on thirty-fourth day from bronchial pneumonia. In one case, dying of heart failure, the treatment was only commenced on the eighth day. Mortality in the fourteen laryngeal cases, counting all the deaths, was 25 per cent., whilst the ordinary mortality in such cases in this hospital is over 50 per cent. Of six cases, with no laryngeal complications, but membrane on tonsils, pharynx, and nares, only one died—five days after the development of scarlatina following the diphtheria—a mortality of 16.6 per cent. After the injections the pulse rate was increased for a few hours; then the pulse diminished in frequency and improved in strength. Membrane disappeared on the average on the ninth day, rather earlier than usual in such severe cases. Bacilli disappeared ordinarily on the nineteenth day, so they were apparently not affected by the treatment. Four cases of post-diphtheritic paralysis occurred, but there were no cases of septic pneumonia.

Dr. Francis Williams[5] has employed antitoxin at the Boston City Hospital in about twenty cases. He does not give mortality statistics, but concludes that in suitable cases the results are wonderfully prompt and good. He advises, if there is no improvement in twenty-four hours, the pulse and temperature being still high, that a second injection should be given.

Dr. Fischer[6] briefly sketches the history of the serum treatment, and he mentions the statistics of German observers, and proceeds to give the details of ten cases, one of which was the case reported by Catlin in the *Medical News*. (See above.) In three cases he injected Aronson's serum in doses of 10 to 20 cc. He treated altogether thirty-four cases, twenty-nine being malignant, four mild, and one moribund. They were not selected cases, varying in all respects as regards general conditions, etc. Of the thirty-four cases there were thirty-two recoveries and two deaths, including the moribund case referred to—a mortality of 5.8 per cent. Injections were made in the intrascapular region. No ill effects were observed from the injections except urticaria. Dr. Fischer states that the temperature does not fall by crisis, but by lysis. He recommends swabbing the membranes with 1:2000 bichloride of mercury solution, and using a nasal douche of warm normal saline solution. In the same number of the *American Journal of Medical Sciences,* Dr. Braden Kyle gives the results of the bacteriological study of four of Dr. Fischer's cases treated with antitoxin at the Municipal Hospital, Philadelphia.

Dr. Porteous[7] cites three cases of a rash appearing after injection of antitoxin. Urticaria frequently occurs, sometimes ill-defined, sometimes well-marked, little or no fever accompanying it. This urticaria is undoubtedly caused by the serum, not necessarily from any poisonous effect, but probably, in some cases at least, from an idiosyncrasy on the part of the recipient. It is said that serum from certain horses is more likely to cause it, but Dr. Porteous thinks there is no proof of this. The amount of serum injected does not seem to account for the effects produced.

Dr. Winslow[8] reports three cases of rash following the injection of antitoxin, one being erythematous, another in the form of a scarlet papular rash, and the third urticarial in its nature.

---

[1]CATLIN. "A case of diphtheria treated with the antitoxin."—*Medical News,* November 10, 1894.

[2]MUEHLECK. "A report of four cases of diphtheria treated by antitoxin."—*Medical and Surgical Reporter,* December 1, 1894.

[3]CAILLÉ. "The treatment of diphtheria with antitoxin—a brief report of nine cases."—*American Medico-Surgical Bulletin,* December 1, 1894.

[4]WHITE. "Antitoxin treatment of diphtheria based upon a series of cases treated at the Willard Parker Hospital."—*American Medico-Surgical Bulletin,* December 1, 1894.

[5]WILLIAMS. "A few cases of diphtheria treated with antitoxin."—*Boston Medical and Surgical Journal,* December 20, 1894.

[6]FISCHER. "Antitoxin in diphtheria in Berlin, New York, and in the Municipal Hospital of Philadelphia."—*American Journal of Medical Sciences,* January, 1895.

[7]PORTEOUS. "Some of the after effects of antitoxin."—*New York Medical Journal,* January 12, 1895.

[8]WINSLOW. "Notes on antitoxin."—*Boston Medical and Surgical Journal,* January 3, 1895.

—*Medical Chronicle.*

---

### THE DIETETIC TREATMENT OF PHTHISIS.

Loomis (*Practitioner,* No. 311, 1894) makes the following suggestions regarding the dietetic treatment of phthisis:

1. Never take cough mixtures if they can possibly be avoided.

2. Food should be taken at least six times in the twenty-four hours; light repasts between the meals and on retiring.

3. Never eat when suffering from bodily or mental fatigue or nervous excitement.

4. Take a nap or at least lie down for twenty minutes before the mid-day and evening meals.

5. The starches and sugars should be avoided, as also all indigestible articles of diet.

6. As far as possible each meal should consist of articles requiring about the same time to digest.

7. Only eat so much as can be easily and fully digested in the time allowed.

8. As long as possible systematic exercise should be taken to favor assimilation and excretion ; when this is impossible, massage or passive exercise should be undergone.

9. The food must be nicely prepared and daintily served,—made inviting in every way.

The following diet-sheet is suggested for the early stage : On awakening, eight ounces of equal parts of milk and seltzer, taken slowly through half an hour. Breakfast: Oatmeal and cracked wheat with a little sugar and an abundance of cream, rare steak or loin chop with fat, soft-boiled or poached egg, cream toast, half pint of milk, and small cup of coffee. Early lunch : Half pint of milk or small tea-cup of squeezed beef juice with stale bread. Mid-day meal : Fish, broiled or stewed chicken, scraped meat-ball, stale bread and plenty of butter, baked apples and cream, and two glasses of milk. Afternoon lunch : Bottle of Koumyss, raw, scraped beef-sandwich, or goblet of milk. Dinner : substantial meat or fish soup, rare roast beef or mutton, game, slice of stale bread, spinach, cauliflower, fresh vegetables in season (sparingly).—*Univ. Med. Mag.*

---

### SOME OF DR. H. C. WOOD'S FAVORITE PRESCRIPTIONS.

*For atonic dyspepsia:*

℞ Strych. sulph.................................................gr. j.
Acidi nitro. mur.......................................f drams ij (if constipated).
or
Acidi nitric dil.................................f drams ij (if bowels are loose).
Tr. card. comp.
Tr. gent. comp...................................................aa f ounces iij.
Liq. pepsinæ..........................................q. s. ad. f ounces viij.
Misce. Sig.—Dessertspoonful after meals.

*Diarrhea mixture:*

℞ Acidi carbolici........................................minims xxiv.
Tr. opii deodoratæ........................................f drams j.
Bismuthi sub-nitratis........................................ounces ss.
Acaciæ pulv........................................q. s.
Syrupi........................................q. s. ad. f ounces viij.
Misce et ft. emulsio.
Sig.—Dessertspoonful every three hours.

*For chronic constipation:*

℞ Aloin........................................gr xv.

Atrop. sulph........................................gr $\frac{1}{3}$.

Strych. sulph........................................gr j.

Misce et ft. pillulæ No. xxx.

Sig.—Take one pill morning and evening.

*For acute colic:*

℞ Chloroformi........................................f drams ij.
Tr. opii deodoratæ........................................f ounces jss.
Olei cajuputi........................................f drams ss.
Acaciæ pulv........................................q. s.
Syrupi........................................q. s. ad. f ounces viij.
Misce et ft. emulsio.
Sig.—Dessertspoonful every two hours.

*For cardiac dropsy:*

℞ Scillæ pulv.
Digitalis pulv........................................aa gr. viii.
Hydrarg. chlor. mite........................................gr. j.
Misce et ft. pil., No. viii.
Sig.—Take one pill every eight hours.

*For acute cystitis:*

℞ Ext. buchu........................................f ounces ij.
Potassi citrat........................................ounces iij.
Spts. etheris nitrosi........................................ounces ss.
Syrupi........................................q. s. ad. f ounces viiii.
Misce. Sig.—Dessertspoonful every five hours.

—*Medical World.*

